# Novel Single Nucleotide Polymorphisms of the Insulin-Like Growth Factor-I Gene and Their Associations with Growth Traits in Common Carp (*Cyprinus carpio* L.)

**DOI:** 10.3390/ijms151222471

**Published:** 2014-12-04

**Authors:** Xiu Feng, Xiaomu Yu, Jingou Tong

**Affiliations:** 1 State Key Laboratory of Freshwater Ecology and Biotechnology, Institute of Hydrobiology, the Chinese Academy of Sciences, Wuhan 430072, China; E-Mails: fengxiu@ihb.ac.cn (X.F.); xmyu@ihb.ac.cn (X.Y.); 2 University of Chinese Academy of Sciences, Beijing 100049, China

**Keywords:** common carp, *IGF-I*, SNP, growth trait, association study

## Abstract

Insulin-like growth factor-I (*IGF-I*) plays an important role in the growth and development of vertebrates. To study polymorphisms of *IGF-I*, we screened a total of 4555 bp of genomic sequences in four exons and partial introns for the discovery of single nucleotide polymorphism (SNP) in common carp (*Cyprinus carpio*). Three SNPs (g.3759T>G, g.7627T>A and g.7722T>C) in intron 2 and a nonsynonymous SNP (g.7892C>T) in exon 3 were identified in a pilot population including random parents and their progenies. 289 progenies were further genotyped for studying possible associations between genotypes or combined genotypes and growth traits. The results showed that the locus g.7627T>A was significantly associated with body weight and body length, and fish with genotype AA had a mean body weight 5.9% higher than those with genotype TT. No significant associations were observed between genotypes of other loci and growth traits. However, when both g.7627T>A and g.7722T>C were considered, the combined genotype TT/TT was extremely associated with the lowest values of body length and body weight and the highest K value in comparison with other diplotypes (*p* < 0.01). These results suggest that genotype AA at g.7627T>A and its combined genotypes with alleles from another locus have positive effects on growth traits, which would be a candidate molecular marker for further studies in marker-assisted selection in common carp.

## 1. Introduction

With the application of molecular genetic technology in animal production, scientists could achieve accurate and efficient artificial selection to improve important economic traits by using marker-assisted selection (MAS) [1]. Identification of candidate genes or genetic markers associated with the traits of interest is the first step in the MAS program [2]. The study of association between polymorphisms in candidate genes and the traits of interest is a convenient and fast approach to identify gene markers which affect quantitative polygenic traits [3]. The significant association is taken as evidence that the gene is either directly involved in the genetic control of the trait or that the functional polymorphism is sufficiently close to the marker so that the two loci are in linkage disequilibrium [4]. For aquaculture species, growth is an important commercial trait which is influenced by environmental factors and multiple genes across the genome. The somatotropic axis which essentially contains growth hormone (GH), insulin-like growth factors (*IGF-I* and *-II*) and their binding proteins and receptors, is known to play a central role in the regulation of growth in livestock and fish [5]. The associations between growth traits and polymorphisms in candidate genes of somatotropic axis have been investigated in several teleosts, such as gilthead sea bream [6], Arctic charr [3], Atlantic salmon [7], Chinese perch [8] and bighead carp [9].

Insulin-like growth factor-I (*IGF-I*) is a polypeptide hormone that shows similar molecular structure to insulin and plays an important role in the growth and development of vertebrates [10]. A cytosine-adenosine (CA) repeat sequence polymorphism in the *IGF-I* promoter region of humans may influence *IGF-I* production and showed association with myocardial infarction, type 2 diabetes [11], bone mineral density [12], risk of heart failure [13], low birthweight [14] and breast cancer [15]. In swine, the CA repeat polymorphism in intron 1 of *IGF-I* was associated with circulating *IGF-I* concentration, growth and fatness [16]. The associations between single nucleotide polymorphisms (SNPs) in *IGF-I* and growth traits have also been reported in other livestock species, such as chicken [17] and cattle [18]. However, there were few studies on the associations between polymorphisms in *IGF-I* and growth traits in fish. A G-to-T transversion was detected in the *IGF-1* promoter region in Arctic charr, but there was no association between the genotypes and growth traits [3]. The polymorphisms in the 5' flanking region of *IGF-1* were associated with growth traits in largemouth bass [19]. One SNP in the promoter region and two SNP in introns of the *IGF-I* were associated with growth-related traits in farmed Atlantic salmon [20].

Common carp *Cyprinus carpio* L., a member of Cyprinidae, is one of the most widely distributed fish species and has the fifth highest annual aquaculture production of 3.8 million metric tons all around the world [21]. Due to its economic importance, quantitative trait loci (QTLs) for growth traits [22,23] have been identified based on the construction of the genetic linkage map. However, owing to the large genome size (2*n* = 100) of common carp, it is necessary to use large numbers of markers to obtain the precise QTL confidence interval. The association study using SNPs of candidate genes provides a powerful tool to elucidate major mutations that affect growth traits. The common carp *IGF-I*, with the length of complete nucleotide sequence of 14077 bp contains five exons and is mainly expressed in liver [24]. Although a l79-bp insertion/deletion polymorphism in *IGF-I* 3'-flanking region has been known to affect growth-related traits in common carp [25], the association between mutations in *IGF-I* exons and introns and growth traits remains unknown. The objectives of this study include: (i) identifying polymorphisms in four exons and partial introns of *IGF-I*; (ii) investigating the association between these genic mutations and growth traits; (iii) evaluating the potential ability of the function mutation to improve growth traits by marker-assisted selection in common carp.

## 2. Results

### 2.1. Identification and Genotyping of the Polymorphisms

**Figure 1:**
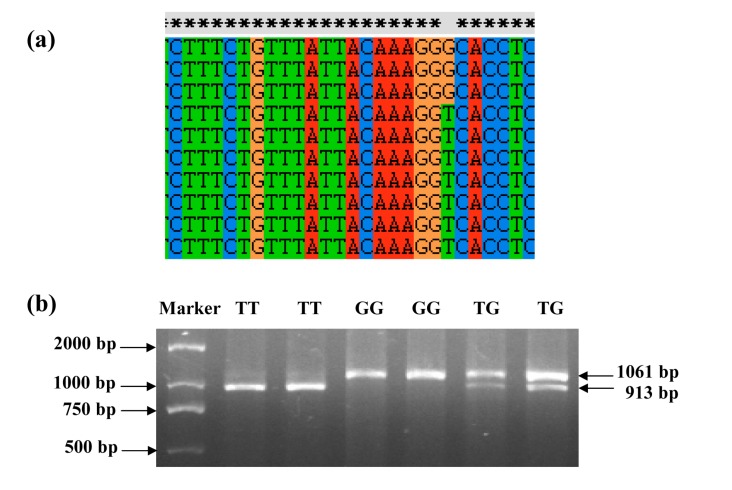
(**a**) Discovery of the single nucleotide polymorphism (SNP) g.3759T>G in intron 2 according to aligned sequences of DNA fragment amplified by the primer pair P3 and (**b**) Electrophoretic patterns for polymerase chain reaction (PCR) fragments of g.3759T>G digested with endonuclease *Bst*EII.

Based on genotyping data of eleven microsatellites (data not shown), the contribution of common carp families to the test population ranged from 0.26% to 10.83%, with an average of 4.35%. Sequences amplified by each primer pair (P1-5) from at least 12 unrelated parents and 5 progenies were aligned to discover polymorphisms in common carp *IGF-I* (GenBank accession No. AF465830.1). In total, nine polymorphic sites were identified, with six SNPs (g.3759T>G, g.7619A>T, g.7620C>T, g.7627T>A, g.7634C>T and g.7722T>C) and one deletion (g.7624delG) in intron 2, one nonsynonymous SNP (g.7892C>T) in exon 3 and one SNP (g.7973C>T) in intron 3. For the locus g.3759T>G, the polymerase chain reaction (PCR) product amplified by primer pair P3 could be digested by endonuclease *Bst*EII to produce 913- and 148-bp fragments for genotype TT, 1061-, 913- and 148-bp fragments for genotype TG, and an 1061-bp fragment for genotype GG (Figure 1). The remaining loci were genotyped by direct sequencing of the PCR products.

### 2.2. Genotypic and Allelic Frequencies

**Figure 2:**
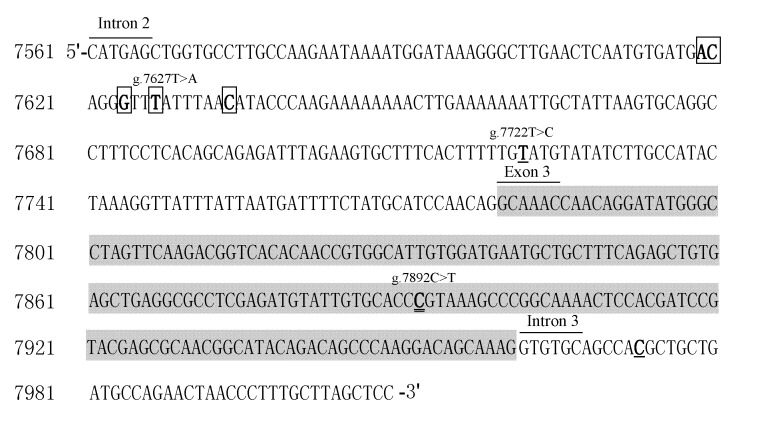
Location of SNPs in *IGF-I* of common carp. Shaded sequence is exon 3, unshaded sequences are introns. SNP sites in boxes are in complete linkage. SNP sites with single underline are also in complete linkage. SNP site with double underlines is a non-synonymous mutation.

Four SNPs (g.7619A>T, g.7620C>T, g.7627T>A, and g.7634C>T) and the deletion g.7624delG were in complete linkage (*r*^2^ = 1) in the tested population of common carp, and other two SNPs (g.7722T>C and g.7973C>T) were also in complete linkage (Figure 2). The loci g.7627T>A and g.7722T>C were analyzed as a representation of the polymorphisms for those loci in complete linkage. The linkage disequilibrium among g.3759T>G, g.7627T>A, g.7722T>C and g.7892C>T was also tested, and these four SNPs were unlinked (*r*^2^ = 0.011–0.254).

The values of genotypic frequencies, allelic frequencies, polymorphism information content (PIC), expected heterozygosity (*H*_E_) and observed heterozygosity (*H*_O_) were shown in Table 1. The *H*_O_ of the four SNPs ranged from 0.059 to 0.308, and the *H*_E_ ranged from 0.083 to 0.501, with the lowest values in g.3759T>G in the tested population of common carp. The estimated PIC value ranged from 0.079 to 0.375, with an average of 0.255. Two SNPs, g.7722T>C and g.7892C>T, significantly deviated from the Hardy-Weinberg equilibriums (HWE) after Bonferroni corrections.

**Table 1:** Frequencies of genotypes and alleles, observed heterozygosity (*H*_O_) and expected heterozygosity (*H*_E_), polymorphism information content (PIC) and test for fitness to Hardy-Weinberg equilibriums (*p*-value) of the SNPs of Insulin-like growth factor-I (*IGF-I*) in the common carp population.

Loci	Allelic Frequency		Genotypic Frequency		PIC	*H*_O_	*H*_E_	*p*-Value
g.3759T>G	T	0.957	TT	0.927	0.079	0.059	0.083	0.071
	G	0.043	TG	0.059				
			GG	0.014				
g.7627T>A	T	0.763	TT	0.637	0.296	0.253	0.301	0.105
	A	0.237	TA	0.252				
			AA	0.111				
g.7722T>C	T	0.486	TT	0.332	0.375	0.308	0.501	0.000 *
	C	0.514	TC	0.308				
			CC	0.360				
g.7892C>T	C	0.799	CC	0.685	0.269	0.228	0.362	0.002 *
	T	0.201	CT	0.228				
			TT	0.087				

* Significant deviation from the Hardy-Weinberg Equilibrium after Bonferroni correction.

### 2.3. Association Analysis of the SNPs with Growth Traits

The locus g.3759T>G was not applied in the association analysis due to its extremely low level of minor genotypic frequency (0.01) and minor allele frequency (0.04). For other three SNPs, only g.7627T>A was significantly associated with body weight (*p* < 0.01) and body length (*p* < 0.01) (Table 2), and individuals with genotype AA had a mean body weight 5.9% higher and a mean body length 2.8% higher than those with genotype TT.

**Table 2:** Association analysis of g.7627T>A with growth traits in common carp.

Genotypes	N	BL (cm)	BW (g)	K
TT	184	18.4 ± 0.9 ^A^	138 ± 14 ^A^	2.21 ± 0.16
TA	73	18.5 ± 1.0 ^A,B^	140 ± 15 ^A,B^	2.19 ± 0.17
AA	32	19.0 ± 0.8 ^B^	146 ± 17 ^B^	2.15 ± 0.13

^A,B^ The different superscript letter within a column means significant difference, *p* < 0.01; The same superscript letter within a column means no significant difference, *p* > 0.05.

Haplotype analysis of the three SNPs revealed that seven haplotypes, H1 (TTC), H2 (TTT), H3 (TCC), H4 (TCT), H5 (ATC), H6 (ATT) and H7 (ACC), were found with haplotype frequencies of 0.284, 0.194, 0.284, 0.002, 0.004, 0.007 and 0.227, respectively. Ten major haplotype combinations were detected, and individuals with the haplotype combination H2H7 had significantly smaller K values than those with H1H3, H1H7 and H3H3 (*p* < 0.05) (Table 3). There were no significant differences among haplotype combinations for body length and body weight.

**Table 3:** Association between haplotype combinations of three SNPs and growth traits in common carp.

Haplotype Combinations	N	BL (cm)	BW (g)	K
H1H1 (TT/TT/CC)	47	18.4 ± 0.8	137 ± 13	2.19 ± 0.14
H1H2 (TT/TT/CT)	24	18.4 ± 0.9	137 ± 15	2.21 ± 0.15
H1H3 (TT/TC/CC)	31	18.3 ± 1.1	137 ± 17	2.26 ± 0.22 ^a^
H1H7 (TA/TC/CC)	15	18.2 ± 1.0	136 ± 13	2.28 ± 0.24 ^a^
H2H2 (TT/TT/TT)	23	18.5 ± 0.8	139 ± 14	2.18 ± 0.13
H2H3 (TT/TC/TC)	22	18.5 ± 0.7	139 ± 14	2.20 ± 0.13
H2H7 (TA/TC/TC)	20	18.8 ± 1.0	140 ± 16	2.10 ± 0.12 ^b^
H3H3 (TT/CC/CC)	40	18.2 ± 0.9	136 ± 15	2.24 ± 0.16 ^a^
H3H7 (TA/CC/CC)	33	18.7 ± 0.8	144 ± 13	2.19 ± 0.14
H7H7 (AA/CC/CC)	30	18.9 ± 0.8	146 ± 18	2.17 ± 0.13

^a,b^ The different superscript letter within a column means significant difference, *p* < 0.05; The same superscript letter within a column means no significant difference, *p* > 0.05.

For the combination of g.7627T>A and g.7722T>C, six major combined genotypes were found, and were significantly associated (*p* < 0.01) with body length, body weight and K values (Table 4). Individuals with the combined genotype TT/TT had significantly higher mean values of body length and body weight and lower mean K values than those with other combined genotypes. Six major combined genotypes were found for the combination of g.7627T>A and g.7892C>T, and individuals with AA/CC had significantly higher values than those of individuals with TT/CC in body length and body weight (*p* < 0.05). For the combination of g.7722T>C and g.7892T>C, no significant difference in three growth traits was observed among individuals with various diplotypes.

**Table 4:** Association between combined genotypes and growth traits in common carp.

Combined SNPs	Combined Genotypes	N	BL (cm)	BW (g)	K
g.7627T>A and g.7722T>C	AA/CC	29	18.9 ± 0.5 ^A^	145 ± 8 ^A^	2.15 ± 0.11 ^A^
	AT/CC	37	19.0 ± 0.6 ^A^	147 ± 10 ^A^	2.13 ± 0.14 ^A^
	AT/CT	36	18.9 ± 0.6 ^A^	146 ± 9 ^A^	2.15 ± 0.12 ^A^
	TT/CC	38	18.9 ± 0.5 ^A^	147 ± 10 ^A^	2.17 ± 0.12 ^A^
	TT/CT	53	18.9 ± 0.5 ^A^	145 ± 11 ^A^	2.15 ± 0.09 ^A^
	TT/TT	93	17.5 ± 0.8 ^B^	125 ± 13 ^B^	2.31 ± 0.18 ^B^
g.7627T>A and g.7892C>T	AA/CC	30	18.9 ± 0.9 ^a^	146 ± 18 ^a^	2.16 ± 0.13
	AT/CC	52	18.5 ± 1.0	140 ± 15	2.23 ± 0.17
	AT/CT	21	18.8 ± 1.0	140 ± 16	2.11 ± 0.13 ^a^
	TT/CC	116	18.3 ± 0.9 ^b^	137 ± 15 ^b^	2.22 ± 0.17 ^b^
	TT/CT	45	18.5 ± 0.8	139 ± 14	2.21 ± 0.14
	TT/TT	23	18.5 ± 0.8	139 ± 14	2.18 ± 0.13

^a,b^ The different superscript letter within a column means significant difference, *p* < 0.05; ^A,B^ The different superscript letter within a column means significant difference, *p* < 0.01; The same superscript letter within a column means no significant difference, *p* > 0.05.

## 3. Discussion

Although *IGF-I* is an excellent candidate gene correlated with growth traits in livestock and fish, few studies on associations between polymorphisms in *IGF-I* and growth traits have been investigated in fish. In this study, four novel SNPs were identified in *IGF-I* of common carp and the associations between SNP genotypes or combined genotypes and growth traits were analyzed.

Under the assumption of Hardy-Weinberg equilibrium for a population, genotype frequency at one locus is a simple function of allele frequencies, and the level of the observed heterozygosity is equivalent to the expected heterozygosity [26]. Significant deviation from HWE based on two SNPs of *IGF-I* in this study may be due to artificial selection and non-random mating [27]. In the test population of this study, g.3759T>G had an extremely low level of minor allele (G) frequency less than 0.05. However, a wild population of common carp from Wuhan, China (*n* = 139) had a frequency of G allele greater than 0.35 at the locus g.3759T>G (data not shown). This may indicate that the genotype TT and allele T were selected largely in the long-term artificial selection process in the test common carp population [28].

The association between genotypes of SNP g.3759T>G and growth traits was not analyzed because the value of the rare genotypes might have been under- or overestimated [29]. The analysis of genetic polymorphisms of other three SNPs of *IGF-I* showed the moderate genetic diversity (PIC values >0.25 and <0.5) in the test population, indicating the important potential for breeding selection [30]. Although the non-synonymous mutation g.7892C>T (changing proline to leucine) was identified in exon 3, no significant association between genotypes and growth traits, indicating that the change of the amino acid residue may do not affect the function and activity of the *IGF-I* in common carp. Only approximately 26%–32% of the natural non-synonymous SNPs have effects on function in human beings [31].

The significant association between g.7627T>A (intron 2) and growth traits may indicate that this mutation is directly involved in the genetic control of the traits or in linkage disequilibrium with a nearby QTL for growth traits [4]. Two intron SNPs associated with growth-related traits have been reported in Atlantic salmon *IGF-I* [20]. Polymorphisms in introns may affect splicing patterns and splicing efficiency, thereby influencing growth traits [32]. One microsatellite in the *IGF-I* have been targeted as a possible QTL marker for birth weight in cattle [33]. Individuals with the dominant genotype TT (a frequency of 63.7%) of the SNP g.7627T>A had negative phenotype values, indicating the direction of artificial selection on breeding at this locus for the test common carp population in this study.

Individual SNPs often provide limited information for testing their associations with traits. By contrast, haplotypes or combinations of SNPs may offer more power to detect associations [34,35,36]. In human, complex diseases may be associated with combinations of SNPs [37]. No significant association was found for g.7722T>C with growth traits in this study, but for the combination of g.7627T>A and g.7722T>C, individuals with combined genotype TT/TT were extremely associated with smaller body length and body weight and higher K value than those of individuals with other combined genotypes (*p* < 0.01). Further studies are required to elucidate this SNP-SNP interaction.

## 4. Experimental Section

### 4.1. Experimental Fish and Phenotypic Data

The common carp population used for association analysis was generated by crossing 30 males and 30 females at a fish farm in Zhenzhou, China. The progenies were hatched at the same time and cultured in a concrete pond following standard culture condition [38]. Body weight (BW) and body length (BL) of 289 randomly collected progenies were measured at the age of nine months. A small piece of fin tissues were cut from each fish and stored at 100% alcohol. Fulton’s condition factor (K) was calculated as K = 100 BW·BL^−3^ [39].

### 4.2. Genomic DNA Extraction

Genomic DNA was extracted from alcohol-preserved fin clips using a traditional proteinase-K digestion and phenol-chloroform protocol [40]. The concentration and purity of the genomic DNA were measured by NanoDrop 2000 spectrophotometer (Thermo Fisher Scientific, Wilmington, DE, USA).

### 4.3. PCR Amplification and DNA Sequencing

**Figure 3:**
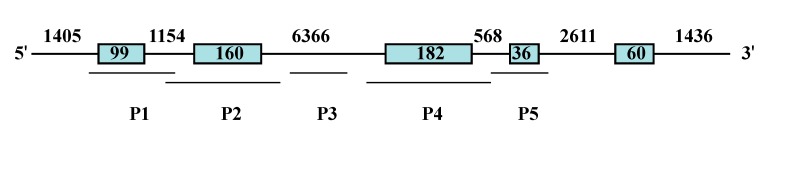
Gene structure of common carp *IGF-I* and location of primer pairs P1–5. Exons are shown as boxes.

Since polymorphisms associated with growth traits have been reported in 5' and 3' untranslated regions (UTRs) of the *IGF-1* in fish [19,20,25], coding sequences of common carp *IGF-I* were screened in this study in the hope to detect nonsynonymous mutations which may alter the structure and function of the protein. Based on the sequence of common carp *IGF-I* (GenBank accession No. AF465830.1), five primer pairs (Table 5) were designed to amplify five DNA fragments which contain four exons and parts of introns (Figure 3). The polymerase chain reaction (PCR) was performed in a 25 μL volume on a veritiTM 96 well thermal cycler (Applied Biosystems, Foster City, CA, USA), with the mixture containing 50 ng genomic DNA, 0.25 μM for each primer, 2.5 μL 10× reaction buffer, 0.5 U Taq DNA polymerase (TaKaRa, Dalian, China), 150 μM of each dNTP and the final volume was adjusted with sterile distilled water. The amplification was programmed for 5 min at 94 °C followed by 37 cycles of 94 °C for 30 s, annealing of 56 °C for 35 s, and 72 °C for 40 s and with a last extension at 72 °C for 15 min. The PCR products were separated on 2.0% of agarose gel and purified using BioSpin Gel Extraction Kit (Biospin, Tokyo, Japan). The purified PCR products were cloned into the PMD 18-T vector (TaKaRa, Otsu, Japan) followed by propagation in *E. coli* DH5α, and then sequenced.

**Table 5:** Primer pairs employed in the study.

Primer Pairs	Primer Sequences (5'–3')	Product Length (bp)	Position
P1	F: CAAATCCGTCTCCTGTTC	822	Exon 1
	R: ATACTGCTGCTTGAACCC		
P2	F: TTGAAGCATACTTGTGCGTTGT	1346	Exon 2
	R: AGTGTGATTGAAGGGAAGGTTT		
P3	F: GCACAATGGCTCAAGGAAGT	1061	Intron 2
	R: GTTTGTATCTGGGGAATGGG		
P4	F: AACTCAATGTGATGACAGGGT	727	Exon 3
	R: GCAACCATCCACCATCTATT		
P5	F: AATAGATGGTGGATGGTTGC	599	Exon 4
	R: CGCTGAGTTTAGAGTTTGGC		

### 4.4. SNP Detection and Genotyping

PCR products from at least 6 sires, 6 dams and 5 progenies were initially cloned and sequenced to detect polymorphisms of common carp *IGF-I* in the test population. Sequences were aligned using Clustal X program [41] for polymorphism identification. The SNPs were genotyped using restriction fragment length polymorphism (RFLP) of the PCR fragments or direct sequencing of PCR products. One SNP within the fragment amplified with primer pair P3 could be genotyped by RFLP using the restriction enzyme *Bst*EII. In brief, the PCR product was digested by *Bst*EII, and then was size-fractionated on 2% of agarose gel and visualized by ethidium bromide staining. The SNPs within the fragment amplified with primer pair P4 were genotyped by direct sequencing of the PCR product using the equencing primer 5'-ATGACAATAACCAAGAGGGC-3'.

### 4.5. Statistical Analysis

ARLEQUIN version 3.1 [42] was used to analyze genetic diversity including genotypic frequencies, allelic frequencies, polymorphism information content (PIC), expected heterozygosity (*H*_E_), observed heterozygosity (*H*_O_) and effective number of alleles (*N*_E_) and test for fitness to the Hardy-Weinberg equilibriums (HWE) of each polymorphic site. The linkage disequilibrium (LD: *r*^2^ = 1, complete linkage and *r*^2^ = 0, no linkage) and haplotype analysis were performed using SHEsis software [43].

The association between genotypes and growth traits were analyzed using the General Linear Model (GLM) of the SPSS 18.0. The model was given as: *Y = μ+ G + e*, where *Y* is the measurement of growth traits, *μ* is mean value, *G* is fixed effect of genotype or haplotype, *e* is random residual error. Because all fish was hatched at the same time and reared in the same pond, and the growth traits were measured at the same age, such factors as breed, site and generation were not considered in this model. Significant differences were tested using Duncan’s multiple range test in the GLM program, and *p* values <0.05 and <0.01 were considered statistically and extremely significant, respectively.

## 5. Conclusions

In conclusion, the present study investigated polymorphisms in *IGF-I* and demonstrated a significant association of g.7627T>A and a combination of g.7627T>A and g.7722T>C with growth traits in the tested population of common carp. The identified SNPs associated with growth performance can be candidate molecular markers in marker-assisted selection programs for improving growth traits in common carp.
